# Biological Treatments in Behçet's Disease: Beyond Anti-TNF Therapy

**DOI:** 10.1155/2014/107421

**Published:** 2014-06-30

**Authors:** Francesco Caso, Luisa Costa, Donato Rigante, Orso Maria Lucherini, Paolo Caso, Vittoria Bascherini, Bruno Frediani, Rolando Cimaz, Edoardo Marrani, Laura Nieves-Martín, Mariangela Atteno, Carmela G. L. Raffaele, Giusyda Tarantino, Mauro Galeazzi, Leonardo Punzi, Luca Cantarini

**Affiliations:** ^1^Interdepartmental Research Center of Systemic Autoimmune and Autoinflammatory Diseases, Rheumatology Unit, Policlinico Le Scotte, University of Siena, Viale Bracci 1, 53100 Siena, Italy; ^2^Rheumatology Unit, Department of Medicine DIMED, University of Padova, Via Giustiniani 2, 35128 Padova, Italy; ^3^Rheumatology Unit, Department of Clinical Medicine and Surgery, University Federico II, Via S. Pansini 5, 80131 Naples, Italy; ^4^Institute of Pediatrics, Cattolica Sacro Cuore University, Largo Agostino Gemelli 8, 00168 Rome, Italy; ^5^La Sapienza University, Viale del Policlinico 155, 00161 Rome, Italy; ^6^Department of Pediatrics, Rheumatology Unit, Anna Meyer Children's Hospital and University of Florence, Viale Pieraccini 24, 50139 Florence, Italy; ^7^Rheumatology Service, Hospital Regional Universitario Carlos Haya, University of Màlaga, Avenida Carlos Haya s/n, 29010 Màlaga, Spain

## Abstract

Behçet's disease (BD) is universally recognized as a multisystemic inflammatory disease of unknown etiology with chronic course and unpredictable exacerbations: its clinical spectrum varies from pure vasculitic manifestations with thrombotic complications to protean inflammatory involvement of multiple organs and tissues. Treatment has been revolutionized by the progressed knowledge in the pathogenetic mechanisms of BD, involving dysfunction and oversecretion of multiple proinflammatory molecules, chiefly tumor necrosis factor- (TNF-) *α*, interleukin- (IL-) 1*β*, and IL-6. However, although biological treatment with anti-TNF-*α* agents has been largely demonstrated to be effective in BD, not all patients are definite responders, and this beneficial response might drop off over time. Therefore, additional therapies for a subset of refractory patients with BD are inevitably needed. Different agents targeting various cytokines and their receptors or cell surface molecules have been studied: the IL-1 receptor has been targeted by anakinra, the IL-1 by canakinumab and gevokizumab, the IL-6 receptor by tocilizumab, the IL12/23 receptor by ustekinumab, and the B-lymphocyte antigen CD-20 by rituximab. The aim of this review is to summarize all current experiences and the most recent evidence regarding these novel approaches with biological drugs other than TNF-*α* blockers in BD, providing a valuable addition to the actually available therapeutic armamentarium.

## 1. Introduction

Behçet's disease (BD) is a chronic and relapsing multisystemic inflammatory disorder which can be localized on the borderline between autoimmune and autoinflammatory diseases [1]. Its incidence is increased around the Mediterranean basin, extending through Middle East and Orient countries, and from a clinical point of view the disorder is mainly characterized by recurrent episodes of mucocutaneous, ocular, joint, vascular, and central nervous system involvement. Recurrent oral and/or genital aphthosis, ocular involvement in terms of uveitis and, retinal vasculitis in combination with variable skin lesions are the cardinal signs of BD [2]. Considerable heterogeneity has been observed among different cohorts of patients with BD, with life-threatening arterial and venous vessel inflammation and thrombotic complications. Furthermore, although somewhat less frequently, BD patients may show joint, gastrointestinal, peripheral, and central nervous system and renal, cardiac, and pulmonary involvement [3]. Its etiology remains still unknown, but the most accredited hypothesis suggests a complex interaction between genetic background and environmental factors, such as microbial agents or their antigens (related to herpes simplex virus, streptococci, staphylococci, or* Escherichia* species) [4]. Human leukocyte antigen (HLA)-B 51, one of the numerous split antigens of HLA-B 5, is the strongest genetic marker of BD in different ethnic groups, as reported both in genome wide association [5, 6] and in meta-analysis studies [7–9]. Although HLA-B 51's mode of action is unclear, antigen presentation ability, molecular mimicry with microbial antigens, or participation in linkage disequilibrium with other genes has been suggested as potential contributive mechanisms in the pathogenesis of BD [7–9]. However, major pathogenetic mechanisms underlying BD are linked to innate immune cell activation and dysregulation, and hyperactivity of neutrophils, T-helper- (Th-) 1, and Th-17 natural killer (NK) cells, the main result of which is the critical overproduction of proinflammatory cytokines, such as tumor necrosis factor- (TNF-) *α*, interleukin- (IL-) 1*β*, IL-6, and IL-17 [10]. Our improved understanding of the molecular mechanisms involved in BD has recently opened up new interesting sceneries in terms of therapy, which might be initiated in the most severely affected patients to avoid complications, such as vascular thrombosis and neurological and/or ocular manifestations [3]. Prior to the introduction of biological agents, options for the treatment of severe BD were limited. In particular, TNF inhibition was successful in controlling inflammation in many patients [11]. However, not all patients responded to different anti-TNF-*α* agents, and loss of efficacy did also appear over time in patients initially responding to anti-TNF biological drugs. Recently many reports have begun to describe BD patients in whom molecular targets other than TNF were sought [12]. The aim of this review is to summarize all current experience and evidence about a new therapeutic biological approach in BD with drugs other than TNF-*α* blockers.

## 2. Cornerstones of Treatment in Behçet's Disease

BD clinical course is highly irregular and erratic, ranging from simple localized mucocutaneous symptoms, that may or may not be associated with uveitis, to severe forms associated with eye and neurological involvement linked to less favourable outcomes. Thus, therapy is mainly based on the type and severity of clinical manifestations and disease duration, as well as number of flares [13]. The mainstay of therapy of isolated aphthosis and acne-like lesions is centred on topical measures [14]. Colchicine at a daily dosage of 1-2 mg/day can be introduced as an additional option in the management of mucocutaneous signs, as its efficacy has been demonstrated in genital aphthosis and erythema nodosum, as well as in joint involvement displayed by female patients [15, 16]. However, data on oral aphthosis and pseudofolliculitis are controversial [15–17], and azathioprine may be considered in cases with severe resistant mucocutaneous and articular involvement [13]. Indeed, azathioprine, usually administered at a daily dosage of 2.5 mg/kg, has been shown to positively impact the long-term prognosis and frequency of mucocutaneous and articular manifestations of BD [18, 19]. Azathioprine importance lies in its beneficial effects on the posterior uveitis [18]. In particular, in a two-year randomized controlled trial in Turkish males with BD, both without and with eye involvement, azathioprine induced a decrease in uveitis flares and protected against the recurrence of uveitis [19]. Thus, its use along with systemic corticosteroids is recommended in BD patients showing eye involvement affecting the posterior segment [13]. In addition to azathioprine, cyclosporine A, at a daily dosage of 5 mg/kg, has also shown its efficacy on the ocular posterior involvement, bringing about improvement in visual acuity during the first 6 months of therapy [20]. Its efficacy at a dosage of 10 mg/kg daily has been demonstrated at a short-term followup, with reduction in both frequency and severity of ocular flares [21]. However, these dosages cannot be considered in long-term treatment due to the risk of secondary nephropathy, hypertension, and neurotoxicity [13]. In addition to azathioprine and cyclosporine A, other immunosuppressive drugs currently used in the management of BD include thalidomide [22], methotrexate [23], and cyclophosphamide [24]. The absence of consolidated data on the efficacy of methotrexate and thalidomide in BD keeps them from being recommended as definite therapeutic strategies [13], although thalidomide has been shown to be potentially useful in the management of severe gastrointestinal involvement prior to implementation of other strategies and surgery [13]. Thalidomide, at the daily dosage ranging from 100 to 300 mg, has also been shown to reduce the frequency of orogenital ulcerations and pseudofolliculitis, but, due to the teratogenic risk and frequent peripheral polyneuropathy, its use is limited [22]. The efficacy of methotrexate, usually employed at a dosage of 7.5–15 mg once a week, has been reported just in one observational study related to posterior uveitis [25]. Efficacy of cyclophosphamide has been proved in patients with ocular, vascular, and neurological involvement [24, 26–30]. With regard to ocular involvement, in a recent study, eye outcomes were evaluated after long-term administration of cyclophosphamide (1 g pulse of cyclophosphamide monthly for 6 months and then every 2-3 months as necessary), azathioprine (at a daily dosage of 2-3 mg/kg), and prednisolone (initiated at 0.5 mg/kg daily and tapered in case of remission) in 295 patients: total adjusted disease activity index significantly improved, but improvement of visual acuity was unremarkable, due to the onset of secondary cataracts [24]. Early use of cyclophosphamide (at a daily dosage of 1 mg/kg given per os or at a dosage ranging from 750 to 1 g/m^2^ every 4 weeks given intravenously) has been considered useful for the vascular complications of BD, including thromboses, occlusions, and large-vessel aneurysms, among the most feared complications due to high potential morbidity and mortality risk [26–29]. Patients with severe neurological clinical signs (meningoencephalitis, dural sinus thrombosis, and severe peripheral nervous system involvement) also require high-dose oral or intravenous corticosteroids in association with cyclophosphamide, at a dosage based on the severity and location of inflammation [30]. For severe and relapsing BD a broad spectrum of therapies consisting of interferon [31] and intravenous immunoglobulins [32] are available, but efficacy data are limited and conflicting [31–33]. To date, therapy has been revolutionized by advances in the knowledge of BD pathogenetic mechanisms, namely, dysfunction and oversecretion of a network of proinflammatory molecules, principally TNF-*α* [10, 34]. Data on anti-TNF-*α* agents are derived from BD case reports and series of patients who were resistant to immunosuppressants and corticosteroids, most of whom suffered from ocular, gastrointestinal, neurological, and vascular manifestations [35–38]. Among anti-TNF-*α* agents, etanercept, a fusion protein of the TNF receptor and IgG_1_ Fc domain, has been shown to reduce the frequency of oral aphthosis and skin lesions combined with a moderate improvement of joint manifestations [35].

Infliximab, a chimeric mouse-human anti-TNF-*α* monoclonal antibody, at a dosage of 5 mg/kg in combination with an immunosuppressive agent, has induced a rapid remission of eye refractory inflammatory signs [39]. Additionally, infliximab, combined with corticosteroids and/or immunosuppressive agents such as cyclosporine A or azathioprine, may be an option in nonemergency cases of gastrointestinal involvement, while its efficacy in patients with parenchymal involvement of the central nervous system is needs to be further evaluated [40–42]. Adalimumab, a humanized IgG_1_ monoclonal anti-TNF-*α* antibody, has been effective in relieving ocular involvement of BD, in particular when patients lost efficacy to infliximab [36].

In the management of gastrointestinal involvement, prior to surgery, sulfasalazine, corticosteroids, azathioprine, thalidomide, and anti-TNF*α* agents should be employed [13]. With regard to ocular involvement, anterior uveitis can be responsive to topical low-dose steroids, while patients with retinal vasculitis, macular involvement, or severe uveitis, defined as a >2-line drop in visual acuity on a 10/10 scale, require azathioprine along with corticosteroids administered orally (prednisone at a daily dosage of 1 mg/kg) or intravenously (methylprednisolone at a daily dosage of 1 g for 3 days), combined with cyclosporine A or infliximab. Corticosteroids, azathioprine, cyclosporine A, and cyclophosphamide are recommended in the management of acute deep vein thrombosis [28, 29]. With regard to the management of central nervous system involvement, corticosteroid therapy is recommended for dural sinus thrombosis, while a combination therapy of corticosteroids with azathioprine, cyclophosphamide, methotrexate, anti-TNF-*α* agents, and interferon may all be considered in cases of meningoencephalitis [13].

## 3. Rationale and Methods

**Figure 1:**
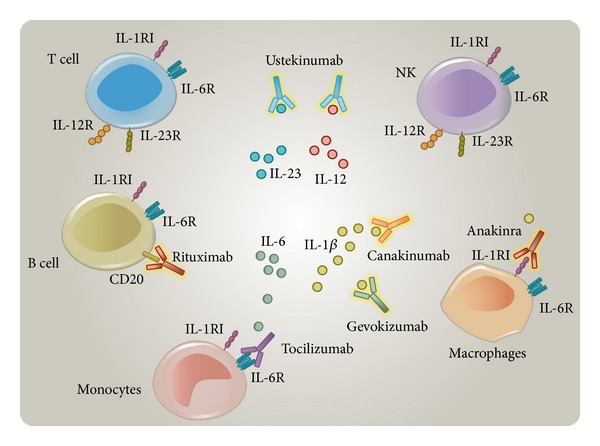
Mechanisms of actions of anakinra, canakinumab, gevokizumab, tocilizumab, ustekinumab, and rituximab, based on the different mechanisms of antagonizing cytokine receptors, cytokines, and cellular antigens.

There is currently no gold standard therapy for BD, and increasing evidence of molecular and cellular pathways involved in its pathogenesis continues to emerge. Recent data have spread the promising therapeutic targets other than TNF in patients with severe and refractory BD (Figure 1). Therefore, we reviewed the available medical literature to find all cases of BD treated with biological agents other than TNF-inhibitors, using the PubMed database. We matched the following search terms: “Behcet's” and “anakinra,” “canakinumab,” “gevokizumab,” “tocilizumab,” “ustekinumab,” and “rituximab,” in order to find studies, including case reports and case series, showing all current experiences and the most recent evidence regarding these novel therapeutic approaches in BD.

## 4. Results

We found 44 cases of BD patients in therapy with biological agents other than anti-TNF-*α* agents. In particular, we found eight studies, describing 24 patients on IL-1 inhibitors [12, 43–50], 13 treated with the IL-1*β* receptor antagonist anakinra [12, 43, 44, 47, 48], 4 with the IL-1 blocker canakinumab [46, 49, 50], and 7, described in one open-label pilot study, with the anti-IL-1 agent gevokizumab [45] (Table 1). Additionally, 7 patients were described being treated with the IL-6 receptor antagonist tocilizumab [51–56], just 1 case with the anti-IL-12/23R agent ustekinumab [57] (Table 2), and 12 with the anti-CD-20 agent rituximab [58–60] (Table 3).

### 4.1. Interleukin-1 Inhibition and Behcet's Disease

The IL-1 superfamily comprises a group of 11 cytokines which regulate many intracellular signaling pathways: IL-1*α* and IL-1*β* are the most studied members, binding their receptor type I (IL-1RI) and coreceptor-accessory protein (IL-1RAcP). While IL-1*α* is expressed as a precursor and is constitutively present in most cells of healthy subjects, IL-1*β*, induced by several cytokines as TNF-*α*, IL-18, IL-1*α*, and IL-1*β* itself, is mainly produced by monocytes, tissue macrophages, fibroblasts, and dendritic cells [61]. IL-1*β* is the principal proinflammatory cytokine, leading to the expression of many chemokines and secondary mediators of inflammation and upregulating innate immunity in response to infectious agents [61]. The inactive precursor of IL-1*β* requires cleavage by an intracellular cysteine protease, called caspase-1, which must be activated to convert IL-1*β* into its bioactive form [61]. The proinflammatory effects of IL-1 are due to the binding with IL-1RI and IL-1RAcP, which together form a heterotrimeric signalling-competent complex; additionally, IL-1*β* autoinduction represents an aspect of the autoinflammation that characterizes many autoinflammatory disorders [62, 63]. IL-1*β* involvement in BD is mainly linked to the evidence of elevated amounts of IL-1*β* in the sera of patients with BD and to the fact that IL-1*β* inhibition has induced a stable clinical remission in different reports [61, 63–65]. Among the available IL-1 blockers, the IL-1 receptor antagonist anakinra, as well as canakinumab and gevokizumab, targeting the IL-1 molecule directly, has been used in patients with BD and provided encouraging preliminary data on the successful IL-1 inhibition, leading to an increased interest in anti-IL-1 agents for managing BD [61, 63]. Anakinra is a recombinant human IL-1 receptor antagonist that competes with IL-1*α* and IL-1*β* and thus inhibits the proinflammatory effects of both cytokines: it has been approved for use in rheumatoid arthritis (at a recommended dose of 100 mg/day subcutaneously) and has been used off-label for a broad spectrum of inflammatory conditions, bringing about a sustained disease remission [61, 63]. In 2008 Botsios et al. reported one BD patient presenting with fever, mucocutaneous involvement, colon ischemic perforation, thrombosis, serositis, and elevated inflammatory markers for whom infliximab was withdrawn due to onset of mucosal abdominal abscesses: anakinra (at the dosage of 100 mg/day) was then started in association with prednisolone (5 mg/day), leading to complete remission in only one week [43]. Two years later Bilginer et al. reported a complete positive response to anakinra (1 mg/kg/day) in a febrile patient diagnosed with familial Mediterranean fever and BD showing mucocutaneous involvement, arthritis, and secondary amyloidosis [44]. Recently, Emmi et al. reported the efficacy of anakinra (100 mg/day) in a patient with mucosal, skin, joint, ocular, and gastrointestinal involvement, in whom a combination of anti-TNF agents and rituximab resulted inefficacious. In this case, a complete positive response was reported at the 12-month followup visit [47]. Additionally, we have recently reported the efficacy of anakinra (100 mg/day) in a patient with BD associated with sacroiliitis, in whom infliximab lost its efficacy despite a concomitant high dosage of prednisone (50 mg/day). Complete remission was verified within a few days, and prednisone was tapered to 5 mg/day without any relapses [48]. Recently, our group has also reported on nine BD patients on anakinra: seven out of nine patients responded to 100 mg/day of anakinra, but two showed no improvement. In six of the seven patients, responses to anakinra were rapid (obtained within 1-2 weeks). Additionally, three out of four patients suffering from recurrent uveitis showed a complete resolution of ocular inflammation. Orogenital aphthosis and skin lesions were the most frequent manifestations refractory to anakinra, with a poor response in seven out of nine patients. In order to control mucocutaneous manifestations, colchicine was successfully introduced in three patients. Thrombotic lesions during treatment with anakinra occurred in two patients, and two others developed retinal vasculitis after 8 months while were on anakinra [12]. In the end, one of two refractory patients achieved complete resolution by increasing the anakinra dose to 150 mg/day.

Canakinumab is a human monoclonal IgG_1_ that selectively neutralizes IL-1*β*, inhibiting its binding to IL-1RI and all cytokine-dependent signaling pathways: the half-life is 21–28 days, and recommended dose is 2 mg/kg subcutaneously in children or 150 mg subcutaneously in adults every 8 weeks. Its safe and successful use has been demonstrated in cryopyrin-associated periodic syndromes and systemic-onset juvenile idiopathic arthritis [61, 63]. Canakinumab administered as monotherapy has also recently been shown to be efficacious in refractory BD, confirming that inhibition of the proinflammatory effects of IL-1*β* is paramount in controlling the clinical spectrum of BD [50]. Additionally, our recent study has suggested that canakinumab given every 6 weeks may be a suitable monodrug therapeutic option for BD patients, confirming the prompt resolution of all disease-related clinical manifestations without any adverse event [50]. Just one patient, previously reported in 2012 when on canakinumab at a dosage of 150 mg every 8 weeks [49], relapsed while was on this dosage, requiring a shorter interval between canakinumab administrations [50]: when canakinumab was administered at the same dosage every 6 weeks a successful response was again obtained, with a stable recovery of patient's clinical picture [50]. One of these patients was also unresponsive to anakinra but took advantage from canakinumab with complete resolution of intraocular inflammation, fever, abdominal pain, and headache within 2 weeks from the start of canakinumab [50]. An additional case related to treatment of BS with canakinumab has recently been published by Ugurlu et al. In this report a single dose of 150 mg of canakinumab was effective in inducing a sustained resolution of BD clinical manifestations, even the ocular ones, and in normalizing all inflammation markers within a few weeks, after that infliximab, adalimumab, and anakinra were all ineffective [46].

Gevokizumab is a recombinant humanized anti-IL-1*β* antibody, that modulates IL-1*β* bioactivity by reducing the affinity for its IL-1RI:IL-1RAcP signaling complex [61]: it has recently been evaluated in BD patients with refractory uveitis. Further convincing evidence of IL-1*β* role in BD derives from a trial based on gevokizumab in patients with multiresistant and sight-threatening uveitis: following a single intravenous infusion of gevokizumab (at the dosage of 0.3 mg/kg) there was a rapid complete resolution of intraocular inflammation along with marked improvement in visual acuity within 21 days. In addition, five patients who were retreated with gevokizumab for recurrent uveitis responded to a second dose and maintained their response for several months, despite discontinuation of immunosuppressive agents and without the need to increase steroid dosage [45].

### 4.2. Interleukin-6 Inhibition and Behçet's Disease

IL-6 is a pleiotropic cytokine secreted by various cell types, including T and B lymphocytes, macrophages, osteoblasts, fibroblasts, keratinocytes, and endothelial cells, involved in many immune pathways and playing a pivotal role in the regulation of various immune responses, in the amplification of acute inflammation, and in its progression into relapsing or chronic inflammatory reactions [66]. Increased plasma IL-6 levels have been reported in patients with BD, mainly in those showing evidence of neurologic involvement, suggesting a correlation with disease activity [67]. Tocilizumab is a humanized monoclonal antibody which specifically inhibits IL-6 by competitively blocking the binding site to the IL-6 receptor, definitely approved for patients with rheumatoid arthritis refractory to traditional disease-modifying antirheumatic drugs. However, due to the IL-6 effects on immune system and inflammatory processes, IL-6 antagonism is now considered a potential therapeutic strategy even in various autoinflammatory and autoimmune disorders [68, 69]. Seven BD patients treated with tocilizumab have been reported [51–56]: all presented orogenital manifestations and six of them cutaneous involvement; ocular involvement was reported in four patients [51, 52, 54, 55], and one of these also suffered from optic neuritis [54]. The reported dosage of tocilizumab was 8 mg/kg every 4 weeks [51–53, 55, 56] or, alternatively, 480 mg every 4 weeks [54]. Tocilizumab monotherapy was used in three cases and brought about complete remission in two [51, 54], while in the third it lost efficacy after the third infusion [56]. Efficacy of tocilizumab was also reported in combination with corticosteroids and other drugs in other two patients [52, 53]. In particular, it is noteworthy that complete remission under tocilizumab was reported in combination with high-dose corticosteroids [52, 53]: in the first case prednisone was used in a dose range of 30–60 mg once daily [52], while in the second prednisone was used at the dosage of 1 mg/kg/day in combination with azathioprine; however, in the second case tocilizumab was discontinued after the fourth infusion due to the occurrence of a scrotal abscess due to* Escherichia coli* [53]. Another BD patient with secondary amyloidosis, treated with colchicine and tocilizumab, showed also a complete remission and decreased proteinuria [55]. However, in another patient the combination of tocilizumab and azathioprine was inefficacious in the treatment of mucocutaneous manifestations [56]. Notably, among BD patients successfully treated with tocilizumab, six had failed to respond to anti-TNF agents [51–54, 56] and one of these became resistant to anakinra and other traditional immunosuppressive drugs [54].

### 4.3. Interleukin-12/23 Inhibition and Behcet's Disease

Two studies have shown increased serum levels of IL-12 and IL-23 in BD patients and also descripted a relationship of serum IL-23 levels with ocular inflammatory activity [70, 71]. There is increasing evidence supporting a link between several single nucleotide polymorphisms of non-HLA and HLA genes and susceptibility to BD [10, 72, 73]. In functional terms, IL-12 and IL-23 are linked to the production of IFN-*γ*, which in turn represents a pivotal mediator of inflammation in peripheral tissues (skin, intestinal mucosa, and lung) by means of multiple proinflammatory cytokines, such as TNF-*α* and IL-1*α* [10]. Moreover, IL-12 and IL-23 share a p40 subunit and promote, respectively, Th1 differentiation and Th17 pathway, which are both involved in the pathogenesis of BD. IL-12, secreted by activated peripheral lymphocytes, interacts with the B1 and B2 subunits of the IL-12 receptor on both human T and natural killer cells, while IL-23, secreted by dendritic cells and activated macrophages, binds to IL-12 receptor B1 and IL-23 receptor: both IL-12 and IL-23 have crucial functions in the adaptive and innate immunity [74].

With regard to ustekinumab, a human monoclonal antibody against the common p40 subunit of IL-12 and IL-23 [75], only one case has been reported by Baerveldt et al. [57]: the patient had BD with mucosal, ocular, intestinal, and articular involvement, as well as psoriasis vulgaris and hidradenitis suppurativa, which were successfully controlled by subcutaneous injections of ustekinumab (at the dosage of 45 mg at weeks 0 and 4 and every 12 weeks thereafter) within 3 months without adjunctive immunosuppressive treatment [57].

### 4.4. B Cell Inhibition and Behcet's Disease

Although there is more extensive evidence of T cell involvement in BD, several studies have suggested a possible pathogenetic role of B cells and a potential close interaction between T and B cells [76–79]. Rituximab is a chimeric monoclonal antibody against CD20, a specific B cell differentiation membrane antigen, participating in B cell activation and proliferation [80], administered intravenously and approved for use in lymphomas (375 mg/m^2^/week for four cycles) [80] and rheumatoid arthritis (1 g × 2/infusions, 2 weeks apart, with repeated courses decided on the individual clinical evaluation) [81]. Rituximab off-label use has been increasing in recent years for other immune-mediated diseases [82–84], as well as for BD [58–60].

In a single-blind randomized controlled trial related to 20 patients with refractory BD involving the eye, 10 patients were treated with rituximab (1 g × 2/infusions, 2 weeks apart) and methotrexate (15 mg/week) and 10 with cyclophosphamide (monthly intravenous infusions of 1000 mg), azathioprine (2-3 mg/kg/day), and prednisone (0.5 mg/kg/day): rituximab and methotrexate were found to be more effective than traditional drugs in improving all the most dreadful ocular manifestations [59]. Moreover, another BD patient with retinal vasculitis refractory to azathioprine and corticosteroids and intolerant to etanercept was successfully treated with rituximab (1 g × 2/infusions, 2 weeks apart) [58]. A young female patient with BD, in whom severe orogenital aphtosis, arthritis, and erythema nodosum were recurrent, who was previously refractory to infliximab and etanercept, was started on rituximab (at the dosage of 1 g given intravenously every two weeks) combined with prednisone (15 mg/day), methotrexate (20 mg/week) and colchicine: this treatment was successful after the third rituximab infusion, allowing a progressive reduction in the corticosteroid dosage [60].

## 5. Conclusive Remarks

The final goal in the treatment strategies of BD is to prevent irreversible multisystemic damage: an ideal therapy should be tailored according to the extent and severity of BD heterogeneous clinical manifestations [11, 13]. Because of the possibility of failure of traditional immunosuppressive and anti-TNF agents, there is need for alternative therapeutic tools with other modes of action, particularly for refractory cases of BD. Based on recurrent inflammatory attacks, lack of autoantibodies, and response to IL-1 inhibition in some patients [12], BD could be depicted as a peculiar autoinflammatory disorder; on the other hand, BD shares with the autoimmune diseases the possibility of being treated with immunosuppressive agents, and therapeutic benefit observed in patients treated with interferon supports the hypothesis of a Th1-driven disease [85]. Although BD classification as an autoinflammatory or autoimmune disorder is still a matter of debate [1, 86, 87], the response to specific novel therapies could provide clinical insights into the causal basis of the syndrome. Multiple cytokines likely contribute to BD pathological landscape, and it is doubtful that blocking a single cytokine or a specific cell line will resolve all of the protean disease manifestations [34]. Among the newer therapies studied to date, inhibition of IL-1*β*, IL-6, and CD20 seems to show the best results. Convincing evidence of IL1*β* role in BD derives from a trial of gevokizumab in patients with multiresistant uveitis [45] and from the successful experience with anakinra [12, 43, 44, 47, 48] and canakinumab [46, 49, 50], while the increasing number of published reports of BD patients treated with tocilizumab [51–56] and rituximab [58–60] demonstrates the complex heterogeneous biochemical scenery behind this syndrome. However, the number of patients on these therapies is still low, making it difficult to draw firm and definite conclusions. Therefore, further large controlled studies involving BD patients and longer-term follow-up periods are needed to corroborate these recent observations and confirm the efficacy and safety of these treatments, which provide a valuable addition to the current therapeutic armamentarium in refractory BD.

## Figures and Tables

**Table 1:** Studies reporting on patients with Behçet's disease treated with anti-IL1*β* agents.

First author (reference, year)	*N* pts	Clinical and laboratory features	HLA-B51	Previous biologic agents and causes of withdrawal	Dosage and eventual cotherapies	Followup	Outcome
Botsios (2008) [43]	1	Fever, mucosal involvement, colon ischemic perforation, necrotizing lymphocytic venulitis, thrombosis, serositis, increase of inflammatory markers, and SPR	Negative	IFX: mucosal abdominal abscesses	ANA 100 mg/day + PDN 5 mg/day	20 months	CR and improvement of inflammatory markers in 7–10 days. Disease-free at 20-month followup

Bilginer (2010) [44]	1	Fever, mucosal involvement, EN, arthritis, secondary amyloidosis, increase of inflammatory markers, and SPR overlapping with FMF	NR	None	ANA 1 mg/kg/day	18 months	CR and improvement of inflammatory markers; patient free of clinical symptoms at 18-month followup; however proteinuria gradually increased (from 1,8 to 2,4 g/day)

Gül (2012) [45]	7	Acute posterior or panuveitis and/or retinal vasculitis	NR	None	GEV: 0.3 mg/kg (single infusion)	Up to disease relapse	Improvement in visual acuity from day 1 in 5 out of 7 patients. Complete resolution of retinal findings achieved in 4–21 days (median 14 days). No detailed assessments of extraocular manifestations were performed. Recurrence of folliculitis and oral aphtosis. Median duration of response: 49 days (range: 21–97 days)

Ugurlu (2012) [46]	1	Mucosal involvement, EN, bilateral panuveitis, retinal vasculitis, and SPR	NR	INF-*γ*: fever; IFX and ANA: flares of uveitis; ADA: loss of efficacy	CAN 150 mg (single dose)	8 weeks	CR for 8 weeks; resolution of ocular inflammation and rapid VA improvement.

Emmi (2013) [47]	1	Mucosal and gastrointestinal involvement, arthritis, pseudofolliculitis, and bilateral retinal vasculitis	NR	IFX: ADR (diffuse urticaria with angioedema); ADA and RTX: persistent uveitis	ANA 100 mg/day	12 months	CR after 12 months of followup; rapid and persistent disappearance of joint pain, mucocutaneous and bowel manifestations; VA improvement, clearing of the vitreous opacity and no active retinal inflammation

Cantarini (2013) [12]	9	Mucosal involvement, EN, headache, retinal vasculitis, low-back pain, and increase of inflammatory markers	Positive	ETN and IFX: loss of efficacy	ANA 150 mg/day + PDN 25 mg/day + colchicine 1 mg/day	9 months	PR; PDN was reduced to 7.5 mg/day
		Fever, mucosal involvement, skin lesions, headache, arthritis, abdominal pain, and increase of inflammatory markers	Positive	None	ANA 100 mg/day	19 months	CR at 12-month followup; onset of DVT after 16 months; CR at 28-month followup
		Fever, mucosal involvement, skin lesions, headache, and increase of SAA	Positive	ANA (100 mg/day): inefficacy	ANA 150 mg/day + PDN 25 mg/day	19 months	DVT not resolved with heparin at 6 months; CYC (5 mg/kg/day) was added; CR with reduction of SAA at 18 months
		Mucosal involvement, bilateral panuveitis, retrobulbar optic neuritis, papillophlebitis, headache, arthralgia, DVT, and increase of SAA	Positive	ETN: loss of efficacy	ANA 100 mg/day + PDN 12.5 mg/day	6 months	Flare of panuveitis after 3 months; ANA was withdrawn; CR with ADA (40 mg twice monthly) + MTX (10 mg/weekly) + PDN (25 mg/day)
		Mucosal involvement, bilateral panuveitis, arthralgia, and increase of inflammatory markers	Positive	None	ANA 100 mg/day + AZA 50 mg/day + PDN 7.5 mg/day	8 months	CR after 12 months
		Fever, mucosal involvement, venous thrombosis, arthritis, panuveitis, headache, pseudofolliculitis, and increase of ESR and SAA	Positive	None	ANA 100 mg/day	12 months	Flare of uveitis after 8 months; ANA was increased to 150 mg/day + MTX (15 mg/weekly) + colchicine (1 mg/day); PR at 17 months of followup
		Mucosal involvement, skin lesions, abdominal pain, photophobia, and increase of SAA	Positive	ADA: inefficacy	ANA 2 mg/kg/day + PDN 15 mg/day	9 months	CR at first; relapse after 4 months requiring an increased dosage of ANA (2.5 mg/kg/day); PR after 7 months
		Fever, mucosal involvement, EN, arthritis, anterior uveitis, pseudofolliculitis, and increase of CRP	Positive	None	ANA 100 mg/day + PDN 10 mg/day	6 months	PR; CYC (5 mg/kg/day) was added after 8 months of followup
		Fever, mucosal and gastrointestinal involvement, headache, anterior uveitis, and arthralgia	Positive	ETN and ADA: loss of efficacy	ANA 100 mg/day	9 months	Inefficacy after 8 weeks; CAN (150 mg every 8 weeks) was started with PR after 2 weeks

Caso (2014) [48]	1	Mucosal and ocular involvement, pseudofolliculitis, sacroiliitis, and increase of inflammatory markers	Positive	IFX: loss of efficacy	ANA 100 mg/day + PDN 50 mg/day	12 months	CR in few days; PDN was tapered to 5 mg/day

Cantarini (2012) [49] Vitale (2014) [50]	3	Fever, mucosal involvement, skin lesions, arthritis, abdominal pain, headache, and increase of inflammatory markers and SAA overlapping with granuloma annulare	Positive	SSZ, MTX, CYC, AZA and LFN: inefficacy; ETN: ADR (recurrent urinary tract infections and bacterial endocarditis); IFX: ADR (recurrent urinary tract infections); ANA: ADR (urticarial lesions)	CAN 150 mg every 8 weeks	16 months	CR in few months; DVT after 16 months: heparin was started and CAN dosing interval was shortened to 6 weeks; CR after 6 months of followup
		Fever, mucosal and gastrointestinal involvement, headache, anterior uveitis, and arthralgia	Positive	ETN and ADA: loss of efficacy; ANA: inefficacy	CAN 150 mg every 6 weeks	12 months	CR after few months
		Fever, mucosal involvement, DVT, panuveitis, headache, arthritis, pseudofolliculitis, and increase of ESR and SAA	Positive	ANA: ADR (urticarial skin lesions)	CAN 150 mg every 6 weeks	6 months	CR within few days

Abbreviations: ADA: adalimumab; ADR: adverse reactions; ANA: anakinra; AZA: azathioprine; BD: Behcet's disease; CAN: canakinumab; CYC: cyclosporine; CMO: cystoid macular oedema; CR: complete remission; CRP: C-reactive protein; CS: corticosteroids; DVT: deep venous thrombosis; EN: erythema nodosum; ESR: erythrocyte sedimentation rate; ETN: etanercept; FMF: familial Mediterranean fever; GEV: gevokizumab; HLA: human leukocyte antigen; Ig: immunoglobulin; IFX: infliximab; INF-*γ*: interferon-gamma; LFN: leflunomide; MTX: methotrexate; *N*: number; NR: not reported; pts: patients; PDN: prednisone; PR: partial remission; RTX: rituximab; SAA: serum amyloid-A; SPR: skin pathergy reaction; SSZ: sulfasalazine; VA: visual acuity.

**Table 2:** Studies reporting on patients with Behçet's disease treated with tocilizumab and ustekinumab.

First author (reference, year)	*N* pts	Main BS clinical and laboratory features	HLA-B51	Previous biologic agents with causes of withdrawal	Dosage and eventual cotherapies	Followup	Outcome
Hirano (2012) [51]	1	Mucosal involvement, EN, and uveitis	NR	IFX: loss of efficacy	TCZ 8 mg/kg every 4 weeks	12 months	VA improvement and resolution of EN and genital aphtosis. Partial improvement of oral aphtosis

Shapiro (2012) [52]	1	Mucosal and neurologic involvement, bilateral uveitis, and cutaneous vasculitis	NR	IFX: concomitant onset of IgA nephropathy	TCZ 8 mg/kg every 4 weeks + PDN 30–60 mg/day	7 months	CR after the 2nd infusion; PDN was tapered off; complete resolution of ocular, neurological, and skin manifestations; oral ulcers recurred

Urbaniak (2012) [53]	1	Mucosal and neurologic involvement, EN, DVT, and thrombophlebitis	NR	IFX: worsening of the gait disturbance and relapse of myelitis	TCZ 8 mg/kg every 4 weeks + AZA 150 mg/day + PDN 1 mg/kg/day	8 months	Improvement of clinical signs and symptoms; after the 4th infusion TCZ was discontinued due to a scrotal abscess

Caso (2013) [54]	1	Fever, mucosal involvement, myalgia, bilateral uveitis, optic neuritis, EN, SPR, and increase of inflammatory markers overlapping with refractory pemphigus foliaceus	Positive	IFX and ADA: inefficacy; ANA: loss of efficacy	TCZ 480 mg every 4 weeks	14 months	CR with improvement of inflammatory markers within few days

Redondo-Pachón (2013) [55]	1	Mucosal involvement, EN, iridocyclitis, secondary amyloidosis, and increase of CRP	Positive	None	TCZ 8 mg/kg every 4 weeks + colchicine 1 mg/day	12 months	CR with decrease of proteinuria and CRP after the 2nd infusion.

Diamantopoulos (2013) [56]	2	Mucosal involvement, pseudofolliculitis, and cutaneous vasculitis	NR	IFX and ETN: short efficacy and ADR (not specified)	TCZ 8 mg/kg every 4 weeks + AZA 150 mg/day	Unknown	Inefficacy; worsening of mouth and genital ulcers
		Mucosal involvement, increase of inflammatory markers	NR	IFX and ADA: incomplete response and ADR (not specified)	TCZ 8 mg/kg every 4 weeks	3 months	Initial PR with loss of efficacy after the 3rd infusion; recurrence of genital ulcers

Baerveldt (2013) [57]	1	Mucosal involvement, anterior uveitis, arthritis, and pathergy reaction overlapping with psoriasis vulgaris and hidradenitis suppurativa	NR	None	Ustekinumab 45 mg at weeks 0 and 4 and every 12 weeks	36 months	CR within few months, clinical improvement of psoriasis

ADA: adalimumab; ADR: adverse reactions; ANA: anakinra; AZA: azathioprine; BD: Behçet's disease; CR: complete remission; CRP: C-reactive protein; DVT: deep venous thrombosis; EN: erythema nodosum; ETN: etanercept; HLA: human leukocyte antigen; IFX: infliximab; MRI: magnetic resonance imaging; MTX: methotrexate; *N*: number; NR: not reported; pts: patients; PDN: prednisone; PR: partial remission; TCZ: tocilizumab; VA: visual acuity.

**Table 3:** Studies reporting on patients with Behçet's disease treated with anti-CD20 monoclonal antibody (rituximab).

First author (reference, year)	*N* pts	Main BD clinical and laboratory features	HLA-B51	Previous biologic agents and causes of withdrawal	Dosage and eventual cotherapies	Followup	Outcome
Sadreddini (2008) [58]	1	Mucosal involvement, arthritis, posterior uveitis, retinal vasculitis, and chronic renal failure (of unknown origin)	NR	ETN: ADR (fever, urticaria, macular rushes, angioedema, transient new lymphopenia, and positive antinuclear antibody test)	RTX 1 g every two weeks for two doses + PDN 1 mg/kg/day	24 months	CR of retinal vasculitis within few months and PDN was tapered to 5 g/day

Davatchi (2010) [59]	10	Mucosal, ocular, and articular involvement; skin manifestations	NR	None	RTX 1 g every two weeks for two doses + PDN 0.5 mg/kg/day + MTX 15 mg/week	6 months	Improvement of ocular manifestations after 6 months. TADAI significant improvement on RTX. VA improved in two patients, remained unchanged in 1, and worsened in 7. Significant improvement of retinal, disc, and macular oedema in all patients

Zhao (2014) [60]	1	Fever, mucosal involvement, arthritis, EN, leukocytoclastic vasculitis, increase of CRP	NR	IFX: inefficacy; ETN: acute mononeuritis multiplex	RTX 1 g every two weeks for two doses + PDN 15 mg/day + MTX 20 mg/week + colchicine	12 months	CR after the 3rd infusion; improved clinical control of disease activity and reduction in steroids requirements (PDN tapered to 8 mg/day)

ADR: adverse reactions; BD: Behçet's disease; CR: complete remission; CRP: C-reactive protein; EN: erythema nodosum; ETN: etanercept; HLA: human leukocyte antigen; IFX: infliximab; MTX: methotrexate; *N*: number; NR: not reported; pts: patients; PDN: prednisone; RTX: rituximab; TADAI: total adjusted disease activity index; VA: visual acuity.
